# Protective Effects of *Bacillus amyloliquefaciens* 40 Against *Clostridium perfringens* Infection in Mice

**DOI:** 10.3389/fnut.2021.733591

**Published:** 2021-10-21

**Authors:** Zipeng Jiang, Wentao Li, Weifa Su, Chaoyue Wen, Tao Gong, Yu Zhang, Yizhen Wang, Mingliang Jin, Zeqing Lu

**Affiliations:** National Engineering Laboratory of Biological Feed Safety and Pollution Prevention and Control, Key Laboratory of Molecular Nutrition, Ministry of Education, Key Laboratory of Animal Nutrition and Feed, Ministry of Agriculture and Rural Affairs, Key Laboratory of Animal Nutrition and Feed Nutrition of Zhejiang Province, Institute of Feed Science, Zhejiang University, Hangzhou, China

**Keywords:** *Clostridium perfringens*, *Bacillus amyloliquefaciens*, mice, immunity, microbiota, metabolic pathways

## Abstract

This study aimed to investigate the protective effects of *Bacillus amyloliquefaciens* (BA40) against *Clostridium perfringens* (*C. perfringens*) infection in mice. *Bacillus subtilis* PB6 was utilized as a positive control to compare the protective effects of BA40. In general, a total of 24 5-week-old male C57BL/6 mice were randomly divided into four groups, with six mice each. The BA40 and PB6 groups were orally dosed with resuspension bacteria (1 × 10^9^ CFU/ml) once a day, from day 1 to 13, respectively. In the control and infected groups, the mice were orally pre-treated with phosphate-buffered saline (PBS) (200 μl/day). The mice in the infected groups, PB6 + infected group and BA40 + infected group, were orally challenged with *C. perfringens* type A (1 × 10^9^ CFU/ml) on day 11, whereas the control group was orally dosed with PBS (200 μl/day). The results showed that the BA40 group ameliorated intestinal structure damage caused by the *C. perfringens* infection. Furthermore, the inflammatory responses detected in the infected groups which include the concentrations of IL-1β, TNF-α, IL-6, and immunoglobulin G (IgG) in the serum and secretory immunoglobulin (SigA) in the colon, and nitric oxide (NO) production and inducible nitric oxide synthase (iNOS) activity in the jejunum, were also alleviated (*P* < 0.05) by BA40 treatment. Similarly, cytokines were also detected by quantitative PCR (qPCR) in the messenger RNA (mRNA) levels, and the results were consistent with the enzyme-linked immunosorbent assay (ELISA) kits. Additionally, in the infected group, the mRNA expression of *Bax* and *p53* was increasing and the *Bcl-2* expression was decreasing, which was reversed by BA40 and PB6 treatment (*P* < 0.05). Moreover, the intestinal microbiota imbalance induced by the *C. perfringens* infection was restored by the BA40 pre-treatment, especially by improving the relative abundance of *Verrucomicrobiota* (*P* < 0.05) and decreasing the relative abundance of *Bacteroidetes* (*P* < 0.05) in the phyla level, and the infected group increased the relative abundance of some pathogens, such as *Bacteroides* and *Staphylococcus* (*P* < 0.05) in the genus level. The gut microbiota alterations in the BA40 group also influenced the metabolic pathways, and the results were also compared. The purine metabolism, 2-oxocarboxylic acid metabolism, and starch and sucrose metabolism were significantly changed (*P* < 0.05). In conclusion, our results demonstrated that BA40 can effectively protect mice from *C. perfringens* infection.

## Introduction

*Clostridium perfringens*, an opportunistic pathogen that can cause diarrhea and fever in animals and humans, can also be found in raw meat and poultry, in the intestines of animals, and the environment (1). Outbreaks tend to happen in some places that serve large groups of people, such as hospitals, school cafeterias, prisons, because there are various sources of infection, including meat, poultry, and other foods cooked at an unsafe temperature (2). *Clostridium perfringens* outbreaks occur most often in November and December, which are linked to commonly served food such as turkey and roast beef. Besides, people of all ages can get food poisoning from *C. perfringens*, and young children and old people are at higher risks of infection (3). Antibiotics have also been used to control the infection by *C. perfringens* (4). However, the antibiotic-residue positive problem had not been solved due to antibiotic overuse (5). There is an urgent and imminent need to develop novel antimicrobial alternatives to reduce the incidence of *C. perfringens* infection while maintaining human health.

Probiotics are live microorganisms and have beneficial effects on the host, which was administered by the Food and Agriculture Organization (FAO) and WHO in 2001 (6). The prevention or control of infectious diseases is one of the most promising health benefits of probiotics (7) because it can protect the host against the invasion of pathogenic bacteria by producing antimicrobial compounds, including surfactin, iturin, and fengycin (8), stimulating the host immune system development (9). A large number of studies have also shown that some microorganisms, such as *Bacillus* (10, 11), *Lactobacilli* (12), *yeast* (13), could alleviate the severity and damage of intestinal inflammation in animals. *Bacillus amyloliquefaciens* strains is a species closely related to *B. subtilis* (14). Additionally, a large number of studies reported that the *B. amyloliquefaciens* contain strong antibacterial activities and suppress numerous pathogens (fungi and bacteria) (14–16). Furthermore, *B. amyloliquefaciens* were used as a probiotic strain to protect against the *C. difficile* associated with a mouse model (17). Similarly, one trial found that *B. amyloliquefaciens* could alleviate diarrhea in weaned pigs (18). In the field of agriculture, *B. amyloliquefaciens* were wildly used as plant growth-promoting rhizobacteria (PGPR) and biocontrol agents (19, 20). Additionally, in the food industry, *B. amyloliquefacien*s also have been speculated to be potential biopreservatives (21). For *B. subtilis* PB6, probiotic significantly improves the intestinal morphology, growth performance, carcass traits, and inhibit the proliferation of *C. perfringens* (11, 22). The current study, therefore, aimed to investigate the protective effect of *B. amyloliquefacien*s (BA40) against *C. perfringens* in mice and compare its function with the probiotic product PB6, then the results provide the evidence that BA40 could help animals or even humans against the intestinal infection of pathogens (*C. perfringens*) and protect intestinal health.

## Methods and Materials

### Bacterial Strain Preparation

*Bacillus amyloliquefaciens* 40 was isolated from the gastrointestinal tract of a 180-day JinHua pig by our laboratory. The BA40 was preserved in the China Center for Type Culture Collection (CCTCC, NO. M2021535). *Bacillus subtilis* PB6 (ATCC-PTA 6737) and *C. perfringens* (ATCC 13124) were purchased from Kemin Industries Inc. (Des Moines, Iowa, United States) and the Guangdong Microbial Culture Collection Center (Guangzhou, China), respectively. In this experiment, the BA40 and PB6 were cultured in a Luria-Bertani (LB) medium at 37°C in a shaking incubator (120 rpm) under aerobic conditions for 12 h. The *C. perfringens* was cultured in a reinforced clostridium medium (RCM) for 24 h (anaerobic environment). The bacteria were harvested by centrifugation at 4,000 g for 10 min at 4°C, washed three times in phosphate-buffered saline (PBS), and centrifuged at 4,000 g for 10 min, respectively. Finally, a final bacterial concentration of 1.0 × 10^9^ CFU (colony forming units)/ml was obtained.

### Animal Experimental Design

**Figure 1:**
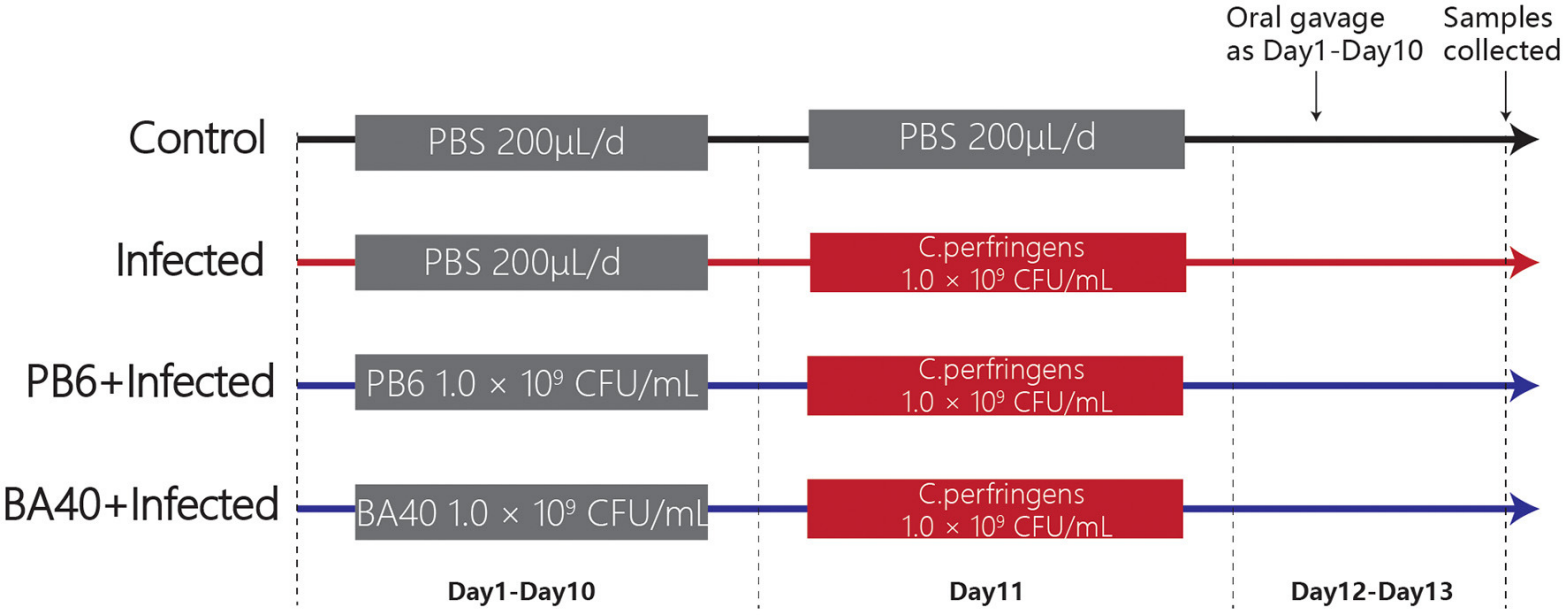
Experimental design and scheme of the animal treatments.

A total of 24 5-week-old male C57BL/6 mice were obtained from the Shanghai Laboratory Animal Co. Ltd. (SLAC, Shanghai, China). Twenty-four mice were randomly divided into four groups (Figure 1) after 1-week adaptation, namely, the control group, infected group, PB6 + infected group, and BA40 + infected group. The PB6 and BA40 group were orally dosed with resuspension bacteria (1.0 × 10^9^ CFU/ml) once a day, from day 1 to 13, respectively. In the control and infected groups, the mice were orally pretreated with PBS. The mice in the infected group, PB6 + Infected group, and BA40 + Infected group were orally challenged with 1.0 × 10^9^ CFU/mL of *C. perfringens* on day 11, whereas the control group was orally dosed with PBS. The mice were weighed every day and were housed in sterile cages containing wood shavings, food pellets, and water. The animal experimental protocol was approved by the Animal Care and Use Committee of Zhejiang University.

### Sample Collection and Treatment

At 12 h after the last gavage, all the mice were weighed and killed. Then, blood samples were collected through cardiac puncture. After centrifugation at 3,000 g for 10 min at 4°C, the serum was prepared. The liver and spleen were weighted and the colon length was measured using a vernier caliper. Simultaneously, the intestines were washed with cold sterile PBS and the collected jejunal samples were prepared for morphology analysis and gene expression determination.

### DLA, DAO, and Inflammatory Cytokines in Serum and Intestine of Mice

The serum levels of D-lactate (DLA) and diamine oxidase (DAO) were quantified with an ELISA kit (Jiangsu Enzyme-Labeled BioTECH, China). Meanwhile, to determine the concentration of IL-1β, IL-6, TNF-α, immunoglobulin A (IgA), and immunoglobulin G (IgG) in the serum, inducible nitric oxide synthase (iNOS) and nitric oxide (NO) in the jejunum and secretory immunoglobulin (SigA) in the colon, ELISA kits (Jiangsu Enzyme-Labeled BioTECH, Yancheng, China) were also used and following the previous study (23). The protocols were carried out according to the instructions of the manufacturer.

### Intestinal Morphology Analysis

To fix the jejunum tissues, 4% paraformaldehyde was used. Afterward, the jejunum tissues were excised, embedded in paraffin, sliced, and stained with hematoxylin and eosin (H&E) (24). Images of the paraffin sections were obtained and observed with a Leica DM3000 Microsystem (Leica, Wetzlar, Germany). To fix the jejunum samples for scanning electron microscopy (SEM) and transmission electron microscopy (TEM) analysis, 2.5% glutaraldehyde was used, following previous studies (25, 26). Then the jejunum samples were fixed in osmic acid and embedded in epon. Digital electron micrographs were obtained with a 1,024 × 1,024 pixel CCD camera system (AMT Corp., Denver, MA).

### RNA Extraction and Quantitative Real-Time PCR

The total RNA was isolated from the jejunum tissues and using a TRIzol regent (Invitrogen Life Technologies, Waltham, Massachusetts, USA). The concentration and purity of the RNA were quantified by measuring its optical density at 260 and 280 nm using a NanoDrop2000 (Thermo Scientific, Wilmington, USA). Then 2 μg of the RNA was used for reverse transcription reaction with random primers. Subsequently, quantitative real-time PCR (qPCR) was conducted in triplicate on a StepOne Real-Time PCR System (ABI StepOnePlus; Applied Biosystems, CA, United States) using a Fast Strat Universal SYBR Green master mix (Roche, Mannheim, Germany), and gene-specific primers (Table 1) were used for the qPCR. The relative messenger RNA (mRNA) expression of the target gene was determined using the 2-ΔΔCt method.

**Table 1:** Primer sequences for qPCR.

**Gene**	**Forward primer sequence (5^′^-3^′^)**	**Reverse primer sequence (5^′^-3^′^)**	**Accession number**
β-actin	CTAGGCGGACTGTTACTGAGC	CGCCTTCACCGTTCCAGTTT	NM_007393.5
IL-1β	GCCACCTTTTGACAGTGATGAG	GACAGCCCAGGTCAAAGGTT	NM_008361.4
IL-6	GACAAAGCCAGAGTCCTTCAGA	TGTGACTCCAGCTTATCTCTTGG	NM_001314054.1
IL-10	CCAAGGTGTCTACAAGGCCA	GCTCTGTCTAGGTCCTGGAGT	NM_010548.2
iNOS	CCTGCAACAGGGAGAAAGCG	TACTGTGGACGGGTCGATGT	NM_001313921.1
TNF-α	ATGGCCTCCCTCTCATCAGT	TTTGCTACGACGTGGGCTAC	NM_013693.3
TGF-β	GTGGCTGAACCAAGGAGACG	GTTTGGGGCTGATCCCGTTG	NM_011577.2
IFN-γ	AAGGAGTCGCTGCTGATTCG	CCGCAATCACAGTCTTGGCT	NM_008337.4
p53	GGGCTGAGACACAATCCTCC	CATTGTAGGTGCCAGGGTCC	NM_001127233.1
Bax	CTGGATCCAAGACCAGGGTG	CCTTTCCCCTTCCCCCATTC	NM_007527.3
Bcl-2	TGAGTACCTGAACCGGCATC	TTGTGGCCCAGGTATGCAC	NM_009741.5
Caspase-3	GCTTGGAACGGTACGCTAAG	CCACTGACTTGCTCCCATGT	NM_001284409.1
Caspase-9	CACCTTCCCAGGTTGCCAAT	CAAGCCATGAGAGCTTCGGA	NM_001277932.1

### Western Blot Analysis of Tight Junction Proteins (TJs)

The total protein of the jejunal mucosa was extracted using a Total Protein Extraction Kit (KeyGen BioTECH, Nanjing, China). The proteins were separated using 10% sodium dodecyl sulfate-polyacrylamide gel electrophoresis (SDS-PAGE) and were electrophoretically transferred onto polyvinylidene difluoride (PVDF) membranes (Millipore, Bedford, MA, USA). The membranes were blocked in tris-buffered saline (TBS) containing 5% fat-free milk and 0.1% Tween 20. Afterward, the proteins were incubated with the primary antibodies overnight at 4°C. After washing with TBS, the proteins were detected with the secondary antibodies for 1.5 h at room temperature. Specific bands were visualized with an enhanced chemiluminescence (ECL) detection kit (KeyGEN BioTECH, Nanjing, China).

### Microbial Analysis of Feces

A QIAamp DNA Stool Mini Kit (QIAGEN Ltd., Hilden, Germany) was used to extract the total genomic DNA from the feces samples which were collected before the mice were slaughtered. The V3-V4 gene region of the bacterial 16S rRNA gene was amplified with the primers 338F (5′-ACTCCTACGGGAGGCAGCAG-3′) and 806R (5′-GGACTACHVGGGTWTCTAAT-3′). A PCR was conducted following a 3 min denaturation at 95°C, 27 cycles of 30 s at 95°C, 30 s for annealing at 55°C, 45 s for elongation at 72°C; and a final extension at 72°C for 10 min. A QIAquick gel extraction kit (QIAGEN, Hilden, Germany) and a Quant-iT PicoGreen dsDNA assay kit (Life Technologies, Carlsbad, United States) were used to further extract, purify and quantify the PCR products.

As the method described by previous studies (27–29), the 16S ribosomal RNA (rRNA) libraries were sequenced on an Illumina HiSeq2500 (Novogene, China). Finally, the data were processed using the QIIME package (V1.7.0, http://qiime.org/scripts/split_libraries_fastq.html) after MiSeq genome sequencing. The sequence data were then deposited in the Sequence Read Archive under the accession number PRJNA730663.

### Statistical Analysis

Statistical tests were performed using Graphpad Prism 8 (San Diego, USA) using one-way ANOVA and a *post-hoc* analysis by the Duncan test. The data are expressed as the mean ± SD. *P* < 0.05 was considered statistically significant.

## Results

### Effect of BA40 Administration on the Growth Performance of the Mice

**Figure 2:**
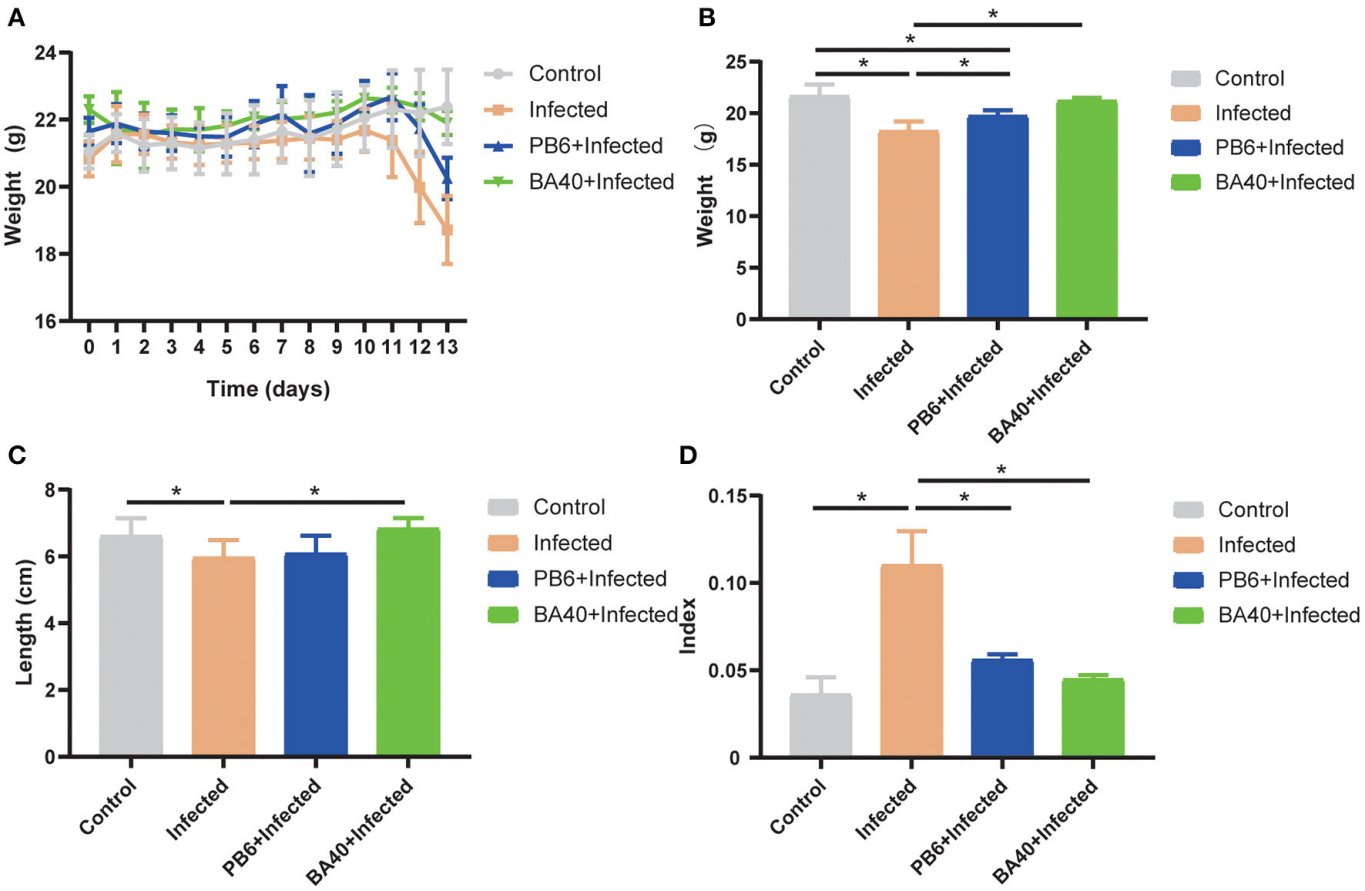
Effect of *Bacillus amyloliquefaciens* 40 (BA40) administration on the growth performance of mice. **(A)** Bodyweight (BW) was weighted among four groups. **(B)** At the end of the experiment, each group was weighted and expressed as mean ± SD. **(C)** The colon length among the four groups. **(D)** The spleen index of the four groups. (*represented significant differences, *P* < 0.05).

The results (Figure 2A) showed that the body weight (BW) of the four groups during the experimental period. After being challenged with *C. perfringens* (ATCC13124), the BW of the infected group was significantly reduced from day 11 to 13, but the BW of the control group and BA40 + infected group had the same trends and almost had no change at all. The BW of the control group, infected group, PB6 + infected group, and BA40 + infected group in day 13 was 21.65 ± 0.46, 18.25 ± 0.38, 19.73 ± **0.2**2, and 21.20 ± 0.12 g, respectively (Figure 2B). Compared with the control group, the BW of the infected group and PB6 + infected group decreased (*P* < 0.05), but the BW of the BA40 + infected group maintain unchanged. As shown in Figure 2C, the colon length did not differ between the control group and the BA40 + infected group (*P* **>** 0.05). The BA40 + infected group showed a significant difference in colon length compared with the infected group (*P* < 0.05). The spleen index among the four groups is presented in Figure 2D and the infected group challenged with *C. perfringens* showed a 2-fold (*P* < 0.05) spleen index more than the other groups.

### BA40 Alleviated Intestinal Mucosal Injury

**Figure 3:**
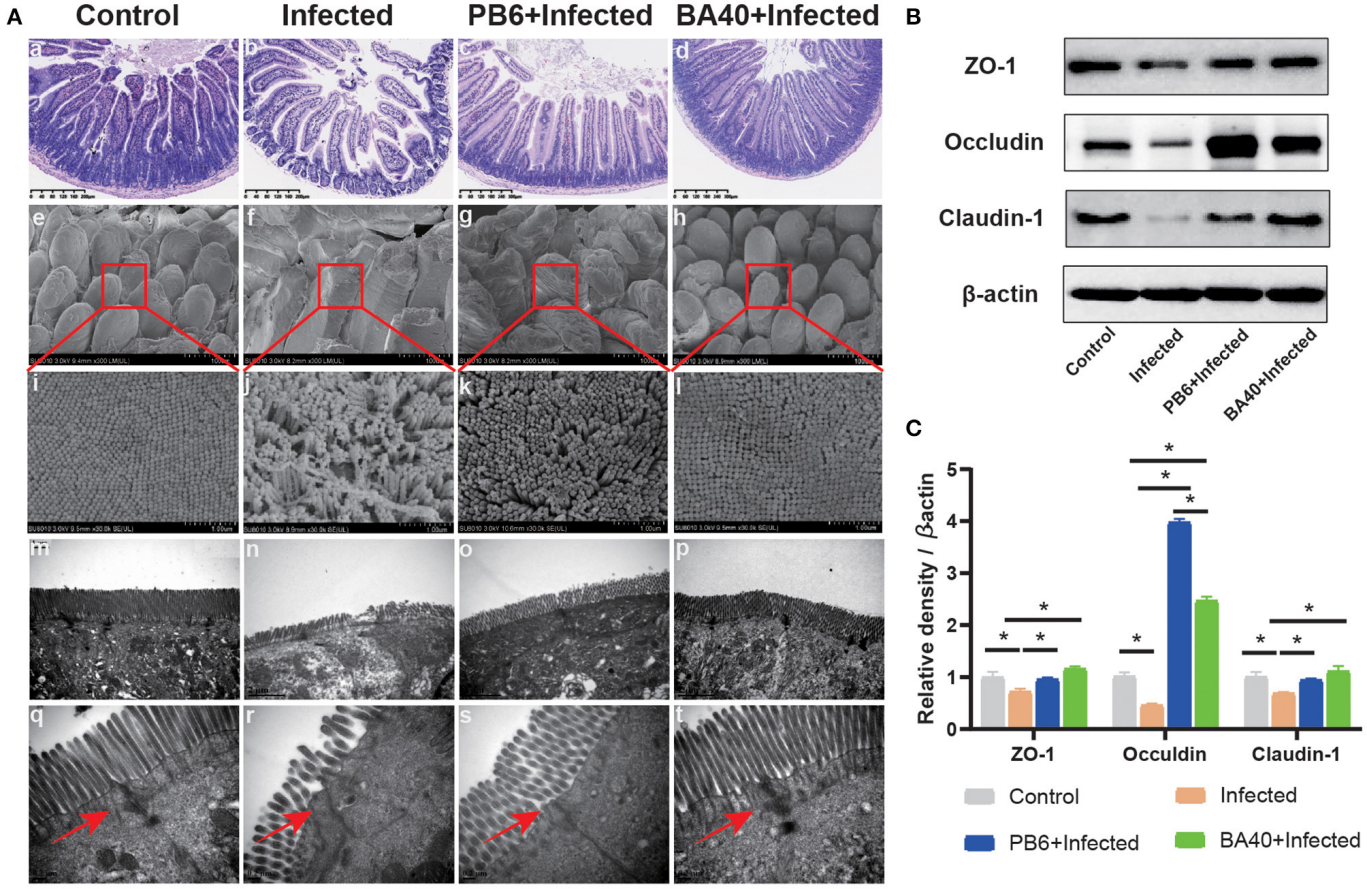
BA40 alleviated intestinal mucosal injury **(A)** Jejunum tissue stained with hematoxylin and eosin (H&E) (bars =300 μm) (a–d). Scanning electron microscopy (SEM) images at × 150 (e–h) and × 30,000 (i–l) fold magnifications. Transmission electron microscopy (TEM) images of the jejunum tissue at × 15,000 (m–p) and × 20,000 (q–t) fold magnifications. **(B)** Western blot analysis of tight junction proteins (TJs) expression. **(C)** The relative levels of TJs quantified by densitometry and normalized to β-actin. (^*^represented significant differences, *P* < 0.05).

To detect the protective effects of BA40 in the *C. perfringens*-induced intestinal injury, Figure 3A shows the jejunum villus morphology analysis. Compared with the control group, the infected group exhibited discontinuous brush edges and blunt villi, which indicate that the villi were injured (Figure 3Ab). However, the pre-treatment with BA40 or PB6 restored the villus morphology (Figures 3Ac,d). Meanwhile, the histopathological evaluation indicated that BA40 presented a protective effect against the *C. perfringens*-induced intestinal injury. Similarly, in the infected group, the SEM images (Figures 3Ae–h) of the jejunum surface showed severe damage, and the BA40 and PB6 pre-treatment alleviated the injury. Moreover, Figures 3Am–p (TEM images of jejunum tissue) indicate that the microvilli under the *C. perfringens* stimulation appeared to be sparse, while the BA40 and PB6 groups possessed neater intestinal microvilli at a scale of 2 μm. Additionally, the tight-junction proteins (TJs) could be observed clearly in the TEM images (Figures 3Aq–t). Compared with the control group, the BA40 group had a similar TJs morphology, which suggests that BA40 could relieve the intestinal microvilli morphology disorder which was challenged by *C. perfringens*. At the molecular level (Figure 3B), the BA40 pre-treatment prevented the decreased expression of the TJs markers ZO-1, Occludin, and Claudin-1 which was induced by the *C. perfringens*. Figure 3C shows the relative levels of TJs expression and normalized to β-actin.

### Effect of BA40 Treatment on DAO, DLA, and Inflammatory Cytokines of Mice

**Figure 4:**
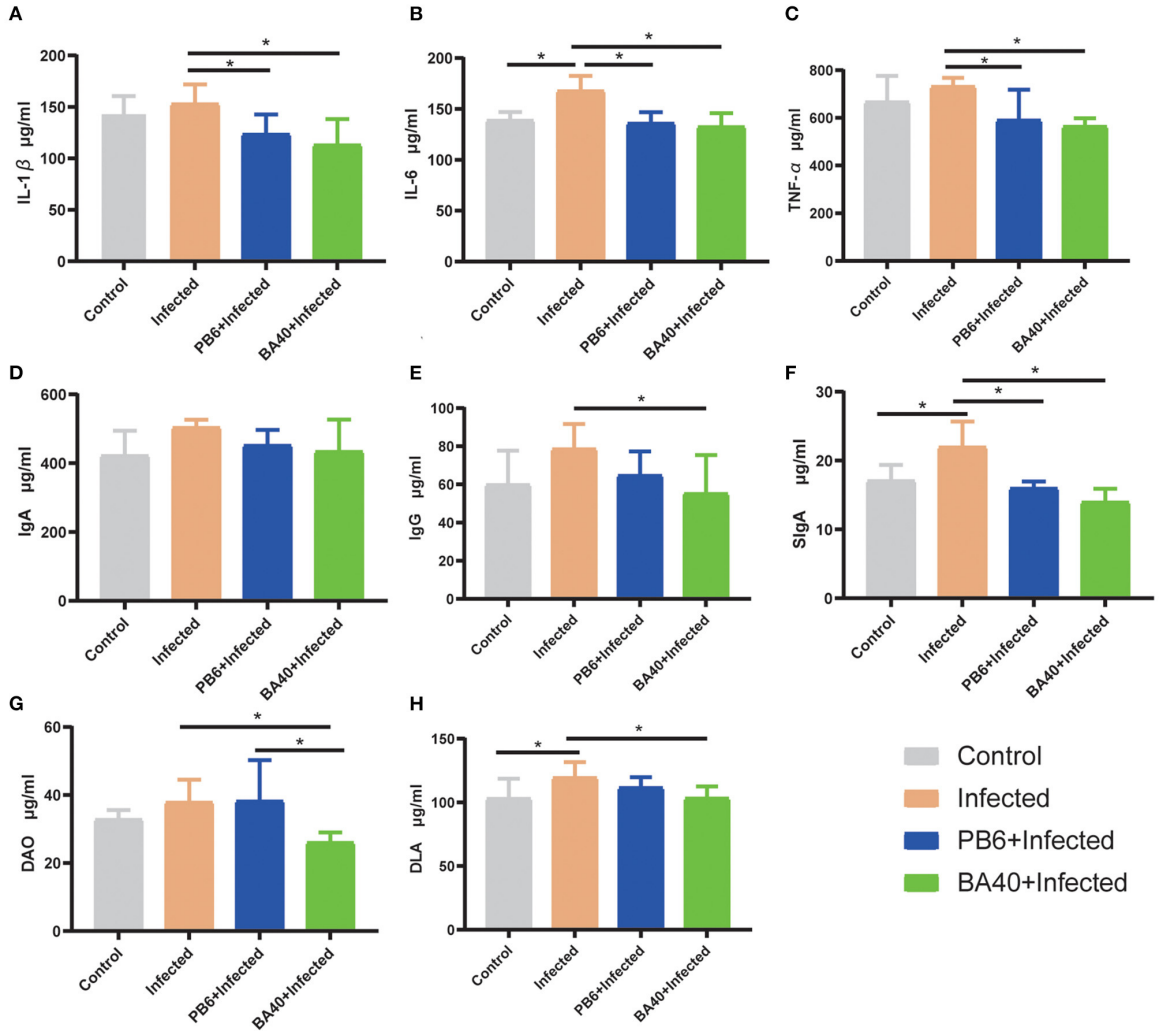
Effect of BA40 treatment on DAO, DLA, and inflammatory cytokines of mice. **(A)** IL-1β (*n* = 6) concentrations. **(B)** IL-6 (*n* = 6) concentrations. **(C)** TNF-α (*n* = 6) concentrations. **(D)** Immunoglobulin A (IgA) (*n* = 6) concentrations. **(E)** Immunoglobulin G (IgG) (*n* = 6) concentrations. **(F)** Secretory Immnoglobulin (SIgA) (*n* = 6) concentrations. **(G)** Diamine oxidase (DAO) (*n* = 6) activity. **(H)** D-lactate (DLA) (*n* = 6) concentrations. Results are presented as mean ± SD (*represented significant differences, *P* < 0.05).

The effect of BA40 on the serum inflammatory cytokines is shown in Figures 4A–E. Compared with the BA40 treatment group, the *C. perfingens* infection increased the concentrations of IL-1β, IL-6, TNF-α, and IgG (*P* < 0.05), while IgA concentrations had no difference (*P* **>** 0.05) among the four groups. The SIgA of the colon tissue had a significant increase (*P* < 0.05) in the infected group compared with the control and BA40 treatment groups (Figure 4F).

Figures 4G,H show the difference of the DAO and DLA concentrations among the four groups in serum level. The DAO and DLA were significantly increased (*P* < 0.05) in the infected group, which indicated that the intestinal barrier was damaged upon being challenged by *C. perfringens*. However, compared with the infected group, the BA40 and PB6 pre-treatment significantly reduced (*P* < 0.05) the DAO concentrations, and the BA40 group also decreased (*P* < 0.05) the DLA concentrations.

### BA40 Down-Regulated Inflammatory Response

**Figure 5:**
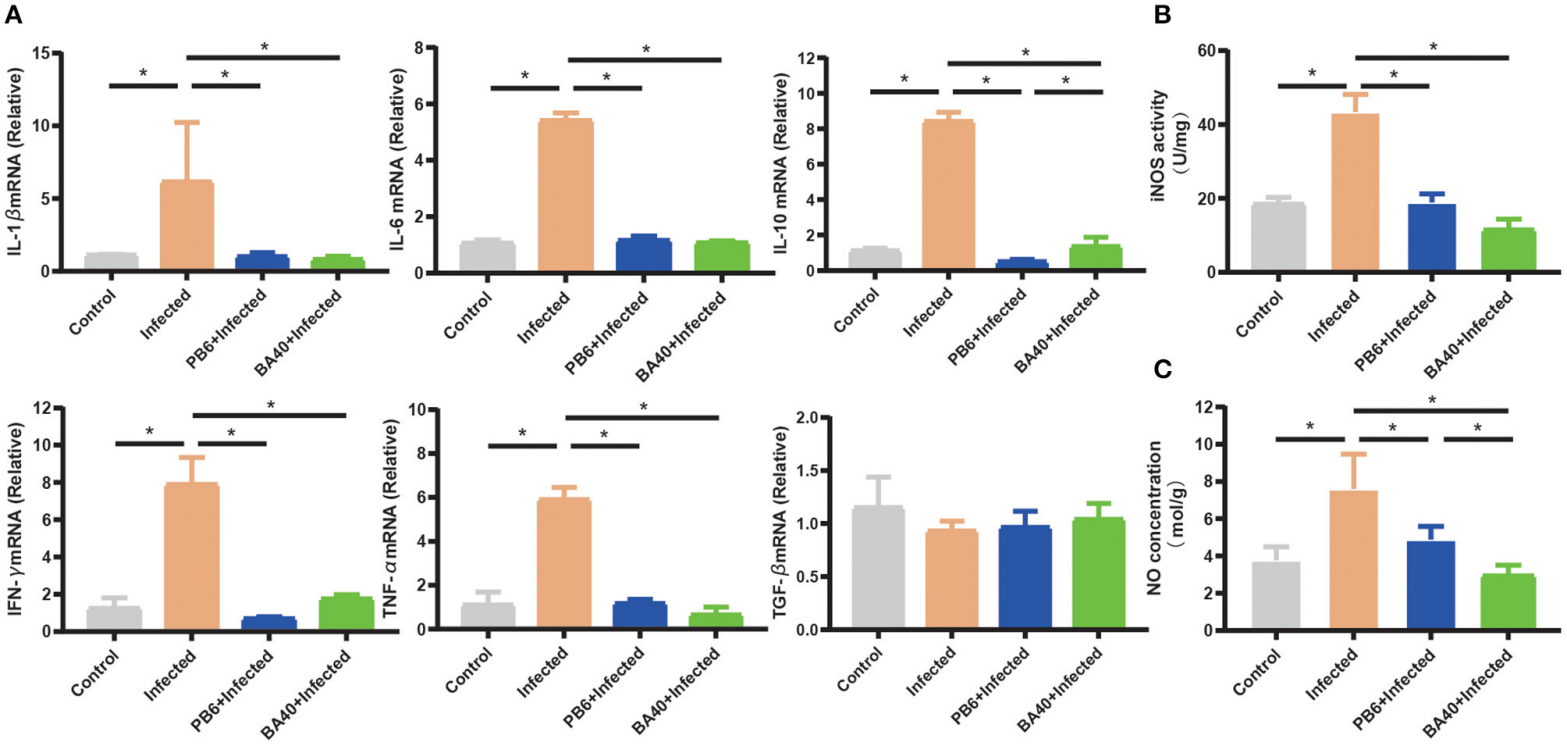
BA40 down-regulated inflammatory response. **(A)** Cytokines gene expression determined by the RT-qPCR. The inducible nitric oxide synthase (iNOS) activity **(B)** and nitric oxide (NO) production **(C)** in the jejunal mucosa were measured using an enzyme-linked immunosorbent assay (ELISA) kit. The results are presented as mean ± SD (*n* = 6/group, *represented significant differences *P* < 0.05).

The anti-inflammatory cytokines *IL-10* and *TNF-*α, and pro-inflammatory cytokines, *IFN-*γ, *IL-1*β, and *IL-6* were increased (*P* < 0.05) in the infected group (Figure 5A), while the pre-treatment with BA40 and PB6 dramatically decreased (*P* < 0.05) the expression of *IL-6, TNF-*α, *IFN-*γ, *IL-1*β, and IL-10. Previous studies found that NO and iNOS play an important role in the host defense process (30). Figures 5B,C depict the highest NO production and iNOS activity in the infected group, but this decreased after the BA40 and BA40 treatment. And the BA40 group decreased the NO production (*P* < 0.05) and iNOS activity (*P* < 0.05) significantly, compared with the infected group.

### BA40 Attenuated Apoptosis Genes Expression

**Figure 6:**
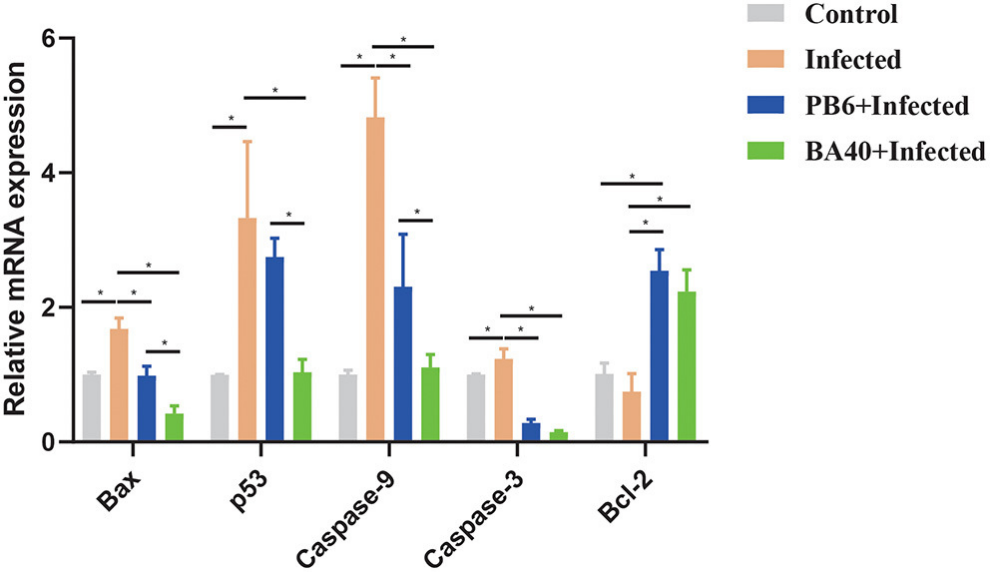
BA40 attenuated apoptosis related gene expression. The apoptosis-related gene expression in the jejunum. The results are presented as mean ± SD (*n* = 6/group, *represented significant differences *P* < 0.05).

The pro-apoptosis genes (*Bax, p53, Caspase-9*, and *Caspase-3*) in the jejunum were upregulated in the infected group (*P* < 0.05). Compared with the infected group (Figure 6), the BA40 treatment downregulated the expression of pro-apoptosis genes significantly (*P* < 0.05), such as *Bax, p53, Caspase-9*, and *Caspase-3*. However, in the PB6 group, there was no similar phenomenon. The BA40 and PB6 group increased the anti-apoptosis gene (*Bcl-2*) expression (*P* < 0.05), and the *Bcl-2* was decreased in the infected group.

### BA40 Reshape the Intestinal Microbiota Composition in *C. perfringens*-Infected Mice

**Figure 7:**
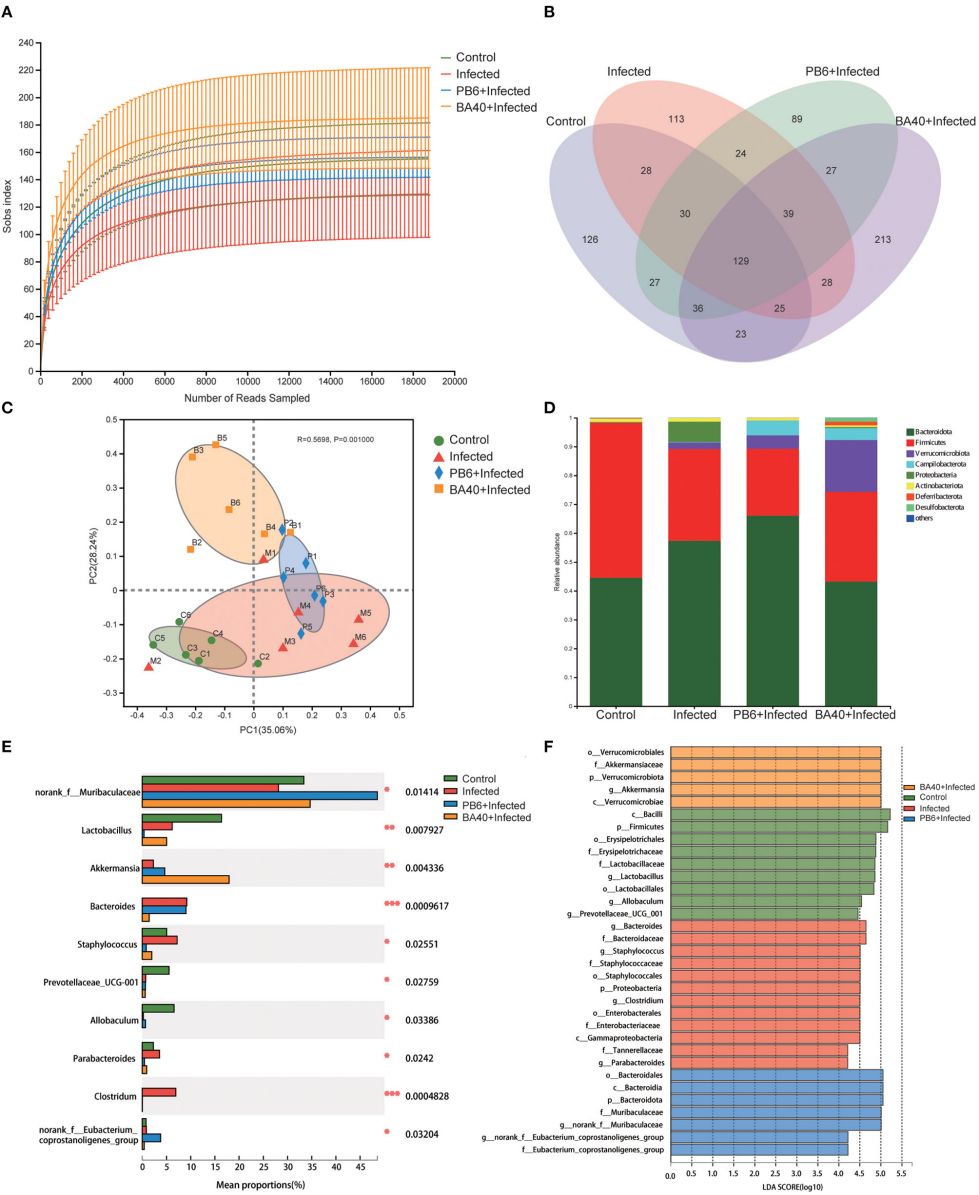
BA40 reshapes the intestinal microbiota composition in *C. perfringens*-infected mice. **(A)** Observed ASV line chart. **(B)** Venn diagram. **(C)** Principal-component (PC) analyses. **(D,E)** Phylum-level **(D)** and genus-level **(E)** of the bacterial community in feces. **(F)** Linear discriminant analysis LDA scores (>4) computed for features at the amplicon sequence variance (ASV) level. Letters represented the taxonomy of the bacteria: p, phylum, c, class; o, order; f, family; g, genus. All results are expressed as mean ± SD in each group (*P < 0.05, **P < 0.01, ***P < 0.01).

The feces microbes were detected using 16s rRNA sequencing technology to investigate the positive effect of the microbiota in the BA40 group under *C. perfringens* stimulation. Overall, 104,809 high-quality sequences were collected by the 16s rRNA sequencing. Additionally, the general 16s rRNA amplicon sequence variant (ASV) numbers reached 625 based on 100% sequence similarity (Table 2). The Good's coverage of all the samples was ~0.99, suggesting that the depth of the sequencing was adequate for the reliable analysis of the bacteria community. Figure 7A shows that the number of the observed ASV in the BA40 group was higher (*P* < 0.05) than in other groups. Figure 7B indicates that the 129 ASVs were shared by all treatments, and the BA40 group had the most unique microbes.

**Table 2:** Characteristics of amplicon libraries in the bacteria community.

	**Data for samples at time(h)**				
**Characteristic**	**Control**	**Infected**	**PB6+ Infected**	**BA40+Infected**	**Total no**.
No. of sequences	27,029 ± 2,430	29,732 ± 5,336	23,859 ± 2,428	24,188 ± 5,489	104,809
No. of ASV	155 ± 28 A	129 ± 34 A	156 ± 16 A	184 ± 40 B	625
Chao1 index	155 ± 29 A	131 ± 35 A	156 ± 16 A	184 ± 41 B	
Shannon index	3.20 ± 0.42 A	2.96 ± 0.28 A	3.21 ± 0.25 A	3.51 ± 0.34 B	
Simpson inedx	0.08± 0.02	0.11 ± 0.04	0.09 ± 0.03	0.09 ± 0.03	

*Means with different letters in each row differ at p < 0.05*.

Furthermore, principal component analysis (PCA) (Figure 7C) was performed and the results show that the four groups were all resolved and distinct, and the BA40 treatment group recovered the microbiota damaged by the *C. perfringens* infection. The results of the PCA plot indicated that BA40 may work more efficiently than PB6 in reshaping the microbial composition. In general, the main microbial compositions in the feces of the four groups were *Firmicutes* and *Bacteroidetes* (Figure 7D). However, the BA40 group was different from the other three groups because it had the highest percentage of *Verrucomicrobiota* which could help the host to maintain the health of the intestine the most among the four groups (31, 32).

In our study, the infected group exhibited a significant increase (*P* < 0.05) in *Proteobacteria* and *Bacteroidetes*, which were effectively relieved by the BA40 (*P* < 0.05). Figure 7E presents the relative abundance of different bacterial generals. The top 10 most abundant generals revealed that the infected group increased the abundance of *Bacteroides* (*P* < 0.001), *Staphylococcus* (*P* < 0.05), *Parabacteroides* (*P* < 0.05) and *Clostridium* (*P* < 0.001), and decreased the *Muribaculaceae* (*P* < 0.05), *Lactobacillus* (*P* < 0.01), *Prevotellaceae* (*P* < 0.05), and *Allobaculum* (*P* < 0.05) abundance in the feces. However, the BA40 group reversed this phenomenon, wherein a decrease of *Staphylococcus* (*P* < 0.05), *Parabacteroides* (*P* < 0.05) and *Clostridium* (*P* < 0.001), *Bacteroides* (*P* < 0.001), and a significant increase of *Akkermansia* (*P* < 0.01) was observed. It appeared that BA40 had a powerful ability to recover the microbiota, especially in restoring the abundance of *Bacteroides* and increasing the abundance of *Akkermansia*. Meanwhile, the results of the linear discriminant analysis (LDA) effect size (LEfSe) showed that there are significant differences in taxonomy (Figure 7F). The dominant species (LDA >4) of the BA40 pre-treatment group were the phylum-level *Verrucomicrobiae* and genus-level *Akkermansia*, which degrades the excess mucus (mucous mucus) produced by the inner walls of the intestine (31). *Akkermansia* is anti-inflammatory and is a protection against obesity, colon cancer, and autism (32). Compared with the infected group, the phylum-level *Proteobacteria*, family-level *Staphylococcaceae*, order-level *Enterobacterales*, and genus-level *Clostridium* dominated the infected group. These bacteria are the main pathogens that endanger human health (33) and the results showed that the sudden increase in the proportion of *Enterobacterales* and *Proteobacteria* could induce intestinal permeability decrease (such as intestinal leakage) and injure intestinal health.

### Bacterial Metabolism of BA40 Treatment

**Figure 8:**
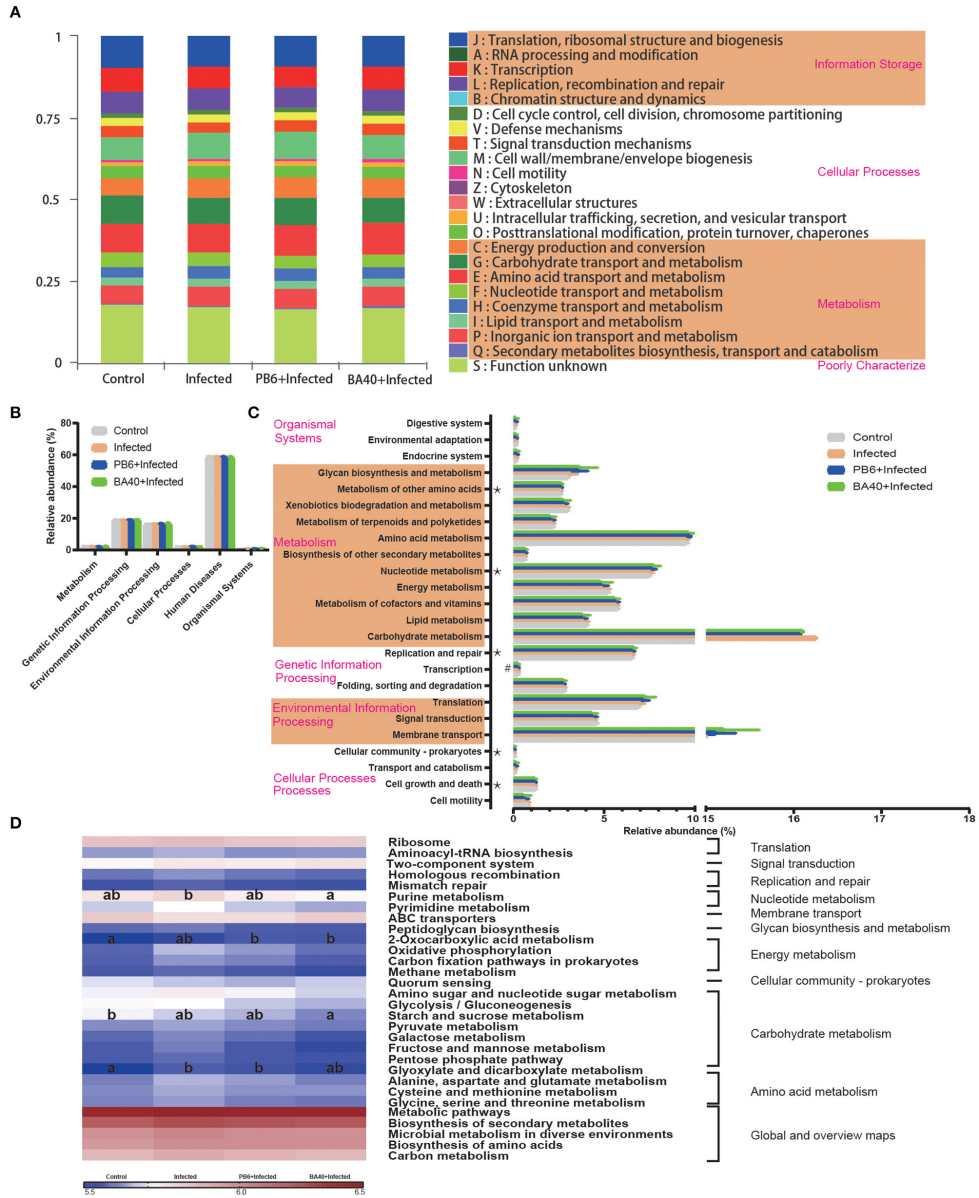
Dynamic of bacterial functional profiles analyzed by PICRUSt (*n* = 6). **(A)** Clusters of orthologous groups of proteins (COG) function classification. **(B)** Metabolic pathways in level 1. **(C)** Kyoto Encyclopedia of Genes and Genomes (KEGG) ortholog functional predictions (Level 2). **(D)** KEGG ortholog functional predictions of the relative abundances of top 30 metabolic functions (Level 3). *Means *P* < 0.05 for BA40 vs. Control and ^#^*P* < 0.0*5* for BA40 vs. PB6. ^a, b^Means with a row with different superscripts significantly differ (*P* < 0.05).

The microbial metabolic function presented in Figure 8 was obtained based on the clusters of orthologous groups of proteins (COG) and the Kyoto Encyclopedia of Genes and Genomes (KEGG) pathway database. Figure 8A shows the changes of the COGs in the four different groups. In the infected group, the information Storage (A), cellular Processes (W), and metabolism (Q) were significantly enhanced (*P* < 0.05). The other functions exhibited no difference (*P* **>** 0.05) among the four groups.

Figure 8B shows the microbial gene function plots of the bacteria in the first level. In the BA40 treatment, the environmental information processing and cellular processes were significantly enriched (*P* < 0.05) compared with the PB6 group. Furthermore, membrane transport and carbohydrate metabolism occupied more than 10% of the enriched pathways in the experimental groups (Figure 8C). Additionally, the sequences related to cell growth and death, folding, sorting, and degradation, transcription, replication and repair, nucleotide metabolism, and the metabolism of other amino acids were significantly enriched (*P* < 0.05) in the BA40 treatment group. Figure 8D presents the third level of the microbial gene functions of bacteria. Amino acid metabolism (glyoxylate and dicarboxylate metabolism) was dramatically enriched (*P* < 0.05) by the *C. perfringens* pre-treatment. In contrast, nucleotide metabolism (purine metabolism), energy metabolism (2-oxocarboxylic acid metabolism), and carbohydrate metabolism (starch and sucrose metabolism) increased with the BA40 treatment.

## Discussion

*Clostridium perfringens* is one of the most common causes of food poisoning. The Centers for Disease Control and Prevention (CDC) estimated that this bacterium causes nearly 1 million illnesses in the United States every year (34, 35). Many studies illustrated that probiotics exert antimicrobial activity using different mechanisms. This study aimed to investigate whether or not BA40 can induce protective effects to prevent *C. perfringens* infection in mice and measured its function by analyzing the intestinal mucosal structure, apoptosis-related gene expression, inflammation response, and intestinal microorganisms. We also compared it with the probiotic product named PB6 and figured out which one has the best preventive effect against *C. perfringens* infection.

The intestinal barrier is mainly composed of three parts, namely, the mucus layer, the epithelial layer, and the underlying lamina propria. The intestinal mucosal barrier is the first line of defense against pathogens (36, 37). The core TJs complex consists of ZOs (Zonula Occludens), occludin, and claudin family members. They connect the intestinal epithelial cells and play the important role of regulating paracellular permeability (38). Intestinal pathogens could impair the intestinal mucosal barrier and cause a systemic inflammatory response, generating a host immune response (39, 40). Many studies have shown that animals (such as piglets, broilers) infected with *C. perfringens* may have highly fatal enteritis and suppressed growth rates (41). Meanwhile, the high levels of DLA and DAO in the serum revealed intestinal barrier injuries (42, 43). Thus, we first evaluated the protective effects of BA40 after the infection. However, our results presented that BA40 protected the gut barrier and maintained the DAO activity and DLA concentrations against the challenge of *C. perfringens*. Moreover, the jejunum villus morphology analysis in the BA40 group illustrated that the BA40 treatment reversed the discontinuous brush borders and blunt villi which only occurred in the infected group. The protective effects were observed clearly in Figure 3, and BA40 was stronger than PB6 in protecting the jejunum against the *C. perfringens* infection. One mechanism revealed that the intestinal epithelial cell apoptosis and shedding by the pathogens may increase gut permeability (44). Furthermore, the results of the H&E, SEM, TEM, TJs structure and the expression of TJs markers (ZO-1, Occludin, and Claudin-1) showed that BA40 effectively attenuated the intestinal barrier dysfunction caused by *C. perfringens* compared with the PB6 group.

Recent studies showed that a possible mechanism of probiotics is the regulation of the immune response; probiotics altered the inflammatory response by stimulating cytokine production (45, 46). Pro-inflammatory cytokines modulate host immunity against many pathogens through multiple mechanisms such as facilitating immune cells differentiation and proliferation, reducing apoptosis, and promoting NO production (47). However, if the immune response is excessive, it could cause tissue injury or damage. For example, *C. perfringens* infection induces a strong inflammatory response and causes tissue damage. Not only can it increase the NO production, iNOS activity, and the mRNA expression of *IL-1*β, *IL-6*, and *IFN-*γ, but also increase the concentration of the cytokines (IL-1β, IL-6, TNF-α, and IgG) in the serum (48). On the other hand, the anti-inflammatory cytokine IL-10 could inhibit immune cells (T cell) proliferation and decrease the host immune response (49). In our study, the IL-10 concentration and expression were decreased by the BA40 pre-treatment, and the plausible explanation was that the BA40 decreased the inflammatory response.

The initiation of cell apoptosis was activated by the imbalance between the Bax (pro-apoptosis proteins) and Bcl-2 (anti-apoptosis protein) (50). A multiple function protein that could also induce apoptosis is p53 (51). Gong et al. (30) studied that *L. plantarum* induces apoptosis by improving the *p53* and decreasing the Bcl-2 in the ilea. In our study, the apoptosis-related genes demonstrated that the probiotics could reform apoptosis induced by *C. perfringens* in the jejunum. It plays a role because the anti-apoptosis genes were mainly upregulated and the pro-apoptosis genes were downregulated. Furthermore, compared with the infected group, the *p53* expression in the BA40 group was downregulated, which indicated that the BA40 could prevent cell apoptosis in the jejunum by the p53 signaling pathway. However, the relative mRNA expression of *p53* and *caspase-9* had no statistical difference between the control and PB6 groups. From the other side, the results indicated that the probiotic functions of BA40 was stronger than PB6.

Recently, gut microbiota has become the most mainstream research object. Gut microorganism exerts critical roles in many aspects, including the immune system, digestion, prevention of enteric pathogen infection, and metabolism function (52). As the report described, probiotic supplementation could modulate the gut microbial community and its relative function (53). *Bacillus amyloliquefaciens* could produce several extracellular enzymes to augment the digestibility and absorption of nutrients in addition to the overall intestinal immune function (54). Additionally, *B. amyloliquefaciens* exerted antagonistic activities against pathogens by producing diverse bioactive metabolites including lipopeptides, fengycin, and iturin (55). One study reported that the fengycin secreted by *B. amyloliquefaciens* could competitively combine the receptor protein accessory gene regulator (AgrC) of bacterial quorum-sensing systems to inhibit the pathogens colonized in animal intestines (8). This process may be applied by BA40 to inhibit the colonization of *C. perfringens* in the small intestine.

Combined with the PCA analysis, the BA40 treatment recovered and improved the microbiota composition damaged by the *C. perfringens* infection. *Firmicutes* and *Bacteroidetes* were reported to be associated with energy efficiency, growth performance, and host health (56). *Akkermansia* is the only member of *Verrucomicrobiota* (phylum) in the gut of mammals and is easy to detect by using 16s rRNA sequencing. A large number of studies reported that the changes of *Akkermansia* in the gut are associated with the health of the host (57–59). *Akkermansia* could ferment mucin and release free sulfate and produces acetate and propionate in the gut, and *Akkermansia* secretes panels of enzymes, such as glycosyl hydrolases, proteases, sulfatases, and sialidases (32).

Additionally, it should be noted that if the proportion of *Proteobacteria* in the gut is higher than others, it can influence the metabolic process, immune system, and imbalance the gut microbial composition (60). The high proportion of *Bacteroides fragilis* also leads to the increased risk of infection and disease (61). *Staphylococcus* is considered to be a disease-causing pathogen in humans and other animals (62). In this study, we used a high-throughput sequencing method based on the 16s rRNA genes and investigated the positive effects of BA40 on the gut microbiota under the challenge of *C. perfringens*. In the infected group, we observed some changes which suggested that the microbial community is in disorder. The increase in the *Proteobacteria* and the decrease in the *Firmicutes*/*Bacteroidetes* ratio were related to the damaged intestinal health. However, the pre-treatment group of BA40 relieved this trend. As for the genus level, we investigated that the *Bacteroides* and *Staphylococcus* declined and *Akkermansia* saw a significant increase between the control group and BA40 group, and BA40 processed a stronger ability in restoring and improving the gut community than PB6. The LDA (score >4) analysis showed that the *Bacteroides, Staphylococcus* and *Enterococcus* et al. made the major contributions in the infected group. However, in the BA40 group, *Akkermansia* became the predominant member of the microbiota community. Kang et al. reported that in dextran sulfate sodium (DSS)-treated mice, the protective role of the extracellular vesicles of *Akkermansia* is evident (63). Previous studies suggest that the abundance of *Akkermansia* decreased significantly in patients and mice with inflammatory bowel disease (64). Our results indicate that BA40 could regulate the abundance of *Akkermansia* which could protect the intestinal health against intestinal inflammation, and the relative abundance of *Akkermansia* may become the biomarker to detect the inflammation response in the gut. The mechanism of *Akkermansia* in protecting the intestinal homeostasis is that it produces acetate and propionate in the gut, which could improve the immune barrier of the host and inhibit the pathogenic substances, such as enterotoxin, to be transferred from the gut to the blood (65). The abundance of *Akkermansia* was increased by the BA40 treatment, so the possible mechanism is that BA40 digests the substrates, then degrades the available polysaccharides which could be applied by *Akkermansia* and promote its proliferation.

The microbiota composition was changed by *C. perfringens* and the metabolic pathways (COG function) were also altered, including the RNA processing and modification, extracellular structures, secondary metabolites biosynthesis, transport, and catabolism, which were reversed by the probiotic pre-treatment. One possible reason for the metabolism function prediction results is that the BA40 improves the digestibility and absorption of nutrients, and the *Akkermansia* used these available polysaccharides to enhance the membrane transport and carbohydrate metabolism function in the BA40 group. Similarly, the results of the KEGG gene function analysis (level 1–3) illustrated that gut microbes affect the host metabolic alterations in health and disease. The purine metabolism, 2-oxocarboxylic acid metabolism, and starch and sucrose metabolism were enriched in the BA40 treatment, while the glyoxylate and dicarboxylate metabolism were enriched in the infected group. For the metabolism function prediction, the BA40 group had better performance than the control group, which increased the starch, sucrose, and 2-oxocarboxylic acid metabolism.

Some reports demonstrated that the diet with the use of *L. gasseri* and low-purine could modulate intestinal purine metabolism. The probiotic supplementation with low-purine diets also improves the host immune system and weakens viral replication, assisting in the treatment of COVID-19 (66). The 2-oxocarboxylic acid metabolism may be related to the mechanism by which the serum growth factors regulate cell multiplication (67). In the BA40 group, we observed that the 2-oxocarboxylic acid metabolism was activated. The starch and sucrose metabolism plays pivotal roles in many biological processes, including the development, stress response, yield formation, and as signals to regulate expression of microRNAs, transcription factors, and for crosstalk with hormonal, oxidative, and defense signaling (68). In the BA40 group, the starch and sucrose metabolism were stronger than the other treatments, mainly through generating sugars as metabolites to fuel growth and synthesize the essential compounds (including protein, cellulose) to support the host as a defense against the invasion of pathogens. In contrast, some studies reported that glyoxylate and dicarboxylate metabolism could become a valuable biomarker to distinguish the liver diseases of the subjects, such as liver cirrhosis (69). In our studies, the infected group activated the glyoxylate and dicarboxylate metabolism and may induce liver injury or damage if the experiment continues for a while.

## Conclusions

To sum it up, the present study demonstrated that the pre-treatment with BA40 exerted the stronger ability compared with PB6 in relieving the *C. perfringens* infection in mice through modulating the intestinal structure, immune response, anti-apoptosis, gut microbiota, and metabolic pathways of microbial composition, indicating that BA40 could be a potential probiotic product for preventing *C. perfringens* infection and protecting the intestinal barrier.

## Data Availability Statement

The datasets presented in this study can be found in online repositories. The names of the repository/repositories and accession number(s) can be found below: https://www.ncbi.nlm.nih.gov/, PRJNA730663.

## Ethics Statement

The animal study was reviewed and approved by Animal Care and Use Committee of Zhejiang University.

## Author Contributions

ZL, MJ, YW, and ZJ conceived and designed the experiment. ZJ analyzed the data and wrote the manuscript. ZJ, WL, and WS carried out the experiment. TG and YZ participated in all laboratory analyses and verified the validity and checked the results. All authors read and approved the final version of this manuscript.

## Funding

The design of the study and collection, analysis, and interpretation of data were supported by China Agriculture Research System of MOF and MARA (CARS-35), Key Agriculture Program of Zhejiang Major Science and Technology Projects (Grant No. 2021C02008, LGN19C170006), Major Scientific and Technological Innovation Projects of Shandong Province of China (Grant No. 2019JZZY020602), and 2020 Talent Cultivation Project by Zhejiang Association for Science and Technology (Grant No. CTZB-2020080127).

## Conflict of Interest

The authors declare that the research was conducted in the absence of any commercial or financial relationships that could be construed as a potential conflict of interest.

## Publisher's Note

All claims expressed in this article are solely those of the authors and do not necessarily represent those of their affiliated organizations, or those of the publisher, the editors and the reviewers. Any product that may be evaluated in this article, or claim that may be made by its manufacturer, is not guaranteed or endorsed by the publisher.
