# Supplementary figures and images for: Protective Effects of Bacillus amyloliquefaciens 40 Against Clostridium perfringens Infection in Mice
Source: Front Nutr. 2021 Oct 21;8:733591. doi: 10.3389/fnut.2021.733591 (PMC8566672; doi:10.3389/fnut.2021.733591)

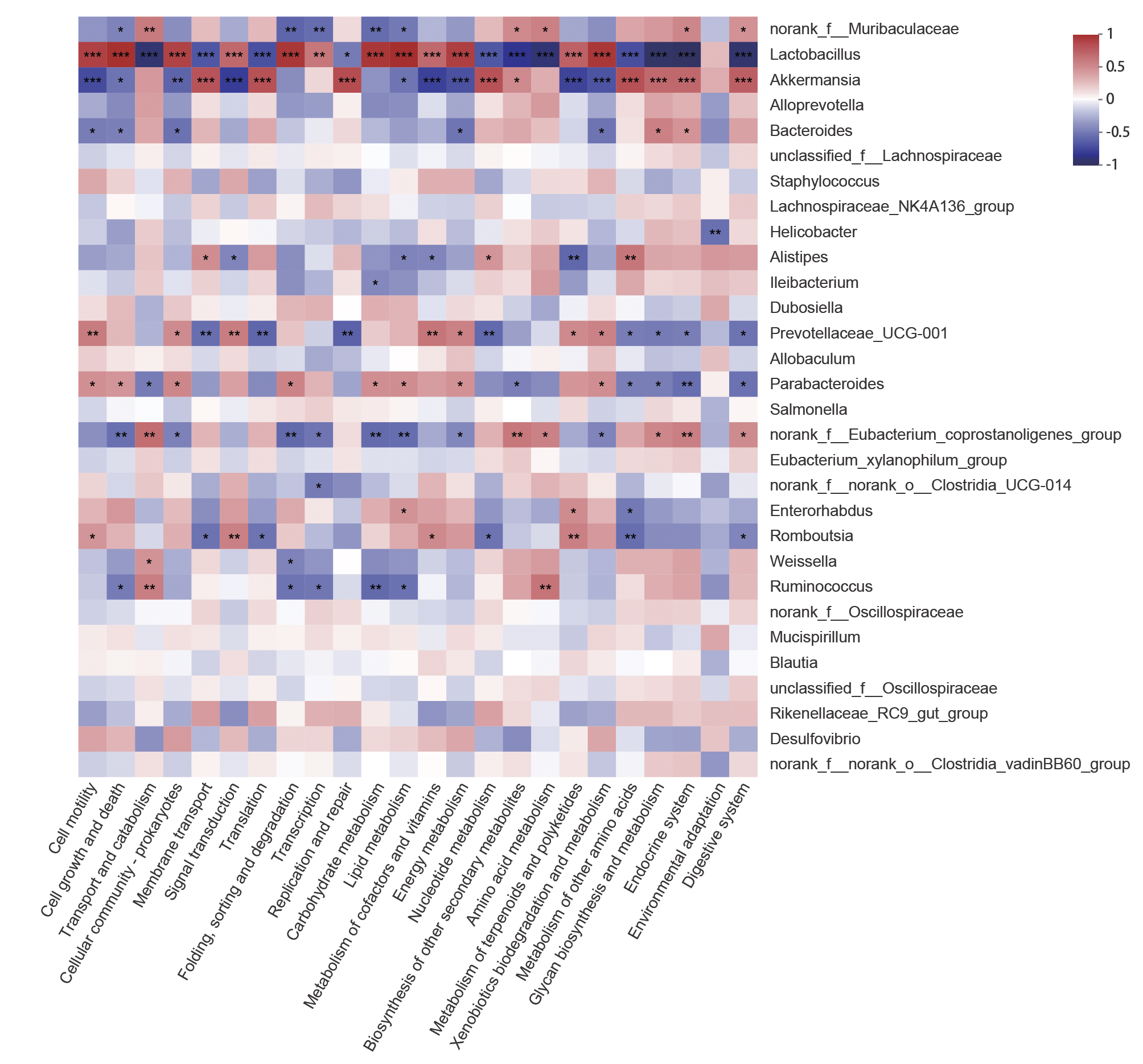

Supplement: Supplementary file 1 [file Image_1.TIF]
